# Development of Application Specific Intelligent Framework for the Optimized Selection of Industrial Grade Magnetic Material

**DOI:** 10.3390/polym13244328

**Published:** 2021-12-10

**Authors:** Muhammad Saleem, Ali Rizwan

**Affiliations:** 1 Department of Industrial Engineering, Faculty of Engineering-Rabigh, King Abdulaziz University, Jeddah 21589, Saudi Arabia; msaleim1@kau.edu.sa; 2 Department of Industrial Engineering, Faculty of Engineering, King Abdulaziz University, Jeddah 21589, Saudi Arabia

**Keywords:** magnetic materials, multivariate statistics, principal component analysis (PCA), descriptive analysis, soft magnetic materials, graph theory, MCDM model

## Abstract

This article attempts to introduce a simple and robust way for the classification of soft magnetic material by using multivariate statistics. The six magnetic properties including coercive magnetic field, relative magnetic permeability, electrical resistivity magnetic inductions, i.e., remanence and saturation along with Curie temperature are used for the classification of 16 soft magnetic materials. Descriptive statistics have been used for defining the prioritization order of the mentioned magnetic characteristics with coercive magnetic field and Curie temperature as the most and least important characteristics for classification of soft magnetic material. Moreover, it has also justified the usage of cluster analysis and principal component analysis for classifying the enlisted materials. After descriptive statistics, cluster analysis is used for classification of materials into four groups, i.e., excellent, good, fair and poor while defining the prioritization order of materials on a relative scale. Principal component analysis reveals that the relative permeability is responsible for defining 99.69% of total variance and is also negatively correlated with the coercive magnetic field. Therefore, these two characteristics are considered the responsible factors for categorically placing the enlisted materials into four clusters. Furthermore, principal component analysis also helps in figuring out the fact that a combined influential consequence of relative permeability, coercive magnetic field, electrical resistivity and critical temperature are responsible for defining prioritization ordering of materials within the clusters. The material’s suitability index is identified while making use of adjacency and decision matrices obtained from material assessment graph and relative importance of magnetic properties, respectively. Afterward this material suitability index is used to rank the enlisted materials based on selected attributes. According to the suitability index, the best choice among enlisted soft magnetic materials is Supermalloy, Magnifer 7904 which is present in group 1 labeled as excellent by multivariate analysis. Therefore, the results of graph theory are in accordance with cluster analysis and principal component analysis, thus confirming the potential of this intelligent approach for the selection application specific magnetic materials.

## 1. Introduction

To maximize the variety of performance metrics, the selection of suitable material for industrial applications is extremely important. However, the selection of magnetic materials from the list of available soft and hard magnetic options is a tedious task, and it requires a smart approach to have an optimum outcome for specific industrial application. Basically, the selection process has three steps: initial monitoring, development and comparison of alternatives, with the final step being finding the best solution. For the selection of suitable soft magnetic material from the list of alternatives, various methods including questionnaires, artificial neural network and Multiple Attributes Decision Making Approaches [1,2,3,4] have been used; however, these methods require special mathematical and computational skills.

Although it is a challenging task to simultaneously consider all the attributes of materials, Ashby [5] successfully demonstrated the feasibility of using multi-objective content option optimization methodology for selecting the suitable magnetic material. The first approach to consider for solving such multi-objective content option optimization problems is the utilizing the concepts of multivariate calculus [6]. However, despite the power of solving complex multi-attribute decision making problems, the applicability of multivariate calculus is not fully explored for sorting magnetic materials. Chauhan and Vaish [7] used the following properties for ranking of soft magnetic materials:

- Curie temperature (Tc, °C);
- Maximum relative magnetic permeability (μ_max_/μ_o_);
- Remanence magnetic induction (Br, T);
- Coercive magnetic field (Hc, A.m^−1^);
- Saturation magnetic induction (Bs, T);
- Electrical resistivity (q, μΩcm).

These properties have been selected due to the relevance in field of application of soft magnetic materials [8,9].

During the study, authors have used the concepts of correlation matrix and hierarchy cluster analysis for supporting the magnetic material ranking obtained from “VlseKriterijumska OptimizacijaI Kompromisno Resenje (VIKOR) and Technique for order preference by similarity to ideal solution (TOPSIS)”. However, a discussion of physical characteristics for ranking outcomes is missing. Furthermore, expertise for understanding the complex mathematical formulation and computer programs is required.

In this study, a simple, robust and systematic way for priority wise listing of physical characteristics and material selection is presented while using the multivariate-statistical analysis. The concepts of coefficient of variance, descriptive analysis, box plots and principal component analysis are utilized for ranking the list of eighteen magnetic materials. The cluster analysis and correlation matrix results reported by Chauhan and Vaish [7] are also revisited to support the prioritization ordering of physical characteristics and classification of materials. In addition to this, graph theory and a matrix approach has been used for the validation of proposed methodology and to choose the best among those listed based on magnetic properties.

### Bakground Literature

According to Cardarelli [8], material selection is critical for product development and design, as well as for the competitiveness and profitability of manufacturers. They have designed a systematic multi-attribute decision making (MADM) model for selecting optimum materials for a Human Powered Vehicle’s fairings. The advantages of modern composite materials over traditional materials with reference to strength to weight ratio are considered in conjugation with other practical factors (e.g., material availability which influences the ultimate choice) for subject matter of interest. Teraiya et al. [10] addressed the problem of connecting rods of I.C. engines while using the five different MADM methods and assessed the limitations/advantages of the chosen decision-making technique. The VIKOR and TOPSIS techniques are identified as the most suitable for focused strategic planning. The accuracy of their finding is substantial for the ability to supervise only those factors and criteria which are important as far as subject matter of interest. Furthermore, VIKOR method’s maximum rank found close to the optimum answer. In another study, Chauhan et al. [11] highlighted the main cause of mechanical malfunction for most components, i.e., wear of mating parts after repeated use. They proved that the MCDM algorithm provides the best solution for solving the selection of suitable coating materials to minimize the wear issue. Similarly, Kumar et al. [12] used MCDM, i.e., AHP and TOPSIS, for a selection of phase-change material (PCM) in thermal control of electronic components and found that AISI A2 steel is the best option for the mentioned application. In Iran’s semi-arid area, two MADM methods have been used to design the best water supply plant. In order to create an efficient production environment, a fuzzy knowledge-based decision analysis approach has been used to choose the best production process and material. Chakrabotry et al. [13] investigated the influence of characteristics loading on the ranking effectiveness of three frequently used MCDM techniques and concluded that the most important criteria with the greatest weight and higher influence on material selection outcomes. Baghel et al. [14] utilized the MCDM for selecting the suitable material for Dye-Sensitized Solar Cells and suggested that the most essential performance factors for a semi-conductor material in DSCs are the budget, static dielectric constant, mobility, band gap and electron injection rate. Zhao et al. [15] used GRA and AHP to develop a material selection analytical framework in the context of green design. In parallel, Gupta et al. [16] used the MADM method with TOPSIS to figure out the best material for the absorber layer of thin film solid cell (TFSC).

## 2. Materials and Methods

A comparatively large number of magnetic materials are present for specific application, but only few of them are useful because of manufacturing process and limitations. Due to the low cost and high Curie temperature, i.e., 850 °C, hard magnetic materials based on Alnico alloys are of great importance. Similarly, ferrites are also in common use because of low cost, high resistivity and wide accessibility. The disadvantage associated with ferrites is their lower efficacy of producing maximum energy. In addition to ferrites, rare earth and transition metals have received much attention because of higher magnetic anisotropy, Curie temperature and coercive field due to their inter metallic phases. Furtherance to it, soft magnetic materials including Si-steels, perm alloys, supermendur, rhometal and Mu-metals are widely used in modern technological applications [15]. The data of six physical characteristics including Tc, μ_max_/μ_o_, Br, Hc, Br, and ρ for the list of eighteen soft magnetic materials are given in Table 1 and have been chosen for the application of multivariate statistics. The enlisted 18 soft magnetic materials and magnetic characteristics were chosen because of their application specific relevancy [16,17].

## 3. Results and Discussion

### 3.1. Hierarchy Cluster Analysis

Cluster analysis is basically a cluster of multivariate databases whose basic function is to provide tools for assembling objects based on individual parametric characteristics. The primary method for the classification of objects in cluster analysis is that each object is comparatively identical to the other with respect to a predefined descriptive criterion. The resulting cluster object findings lead to high-internal homogeneity and high-external heterogeneity to differentiate between important and irrelevant variables. Different methods have been used for clustering data but they are mainly classified into hierarchical and non-hierarchical methods [17,18]. The present study is based on hierarchical clustering which represents materials in a hierarchical structure (dendrogram). The main purpose of this study is to measure a relationship between individual clusters and to classify the similarity and dissimilarity of each material. The dendrograms can be generated using different linkage methods to determine the distance between clusters. An estimation of the cophenetic correlation coefficient will determine the efficacy of a dendrogram. A higher value of this coefficient, closer to one, indicates the accuracy of physical clustering with respect to actual similarities between the selected alternatives [19,20].

### 3.2. PCA or Principal Component Analysis

Principal Component Analysis (PCA) is a statistical method using an orthogonal transformation to convert a set of observations of potentially associated variables into a set of values of linearly uncorrelated variables called principal components. The new axis lies along the maximum variance direction, and there is a new cluster of factors in PCA called the axis rotation which is used to separate the original variables into groups. PCA is primarily used as a tool for exploratory data analysis and predictive models. PCA provides some explanation for the most valuable factor representing the full interpretation of the information set. PCA summarizes the statistical correlation of material’s physical properties with the least reduction in available information. The following equation is used to express the Principal Component Analysis:

Z_ij_ = a_i1×1j_ + a_i2×2j_ + ... + a_im×mj_

where Z is the component score, a is the component loading, x is the measured value of a variable, i is the component number, j is the sample number, and m is the total number of variables [20].

## 4. Results and Discussion

### 4.1. Application of Multivariate Statistics for Magnetic Material Selection

#### 4.1.1. Descriptive Analysis

**Figure 1:**
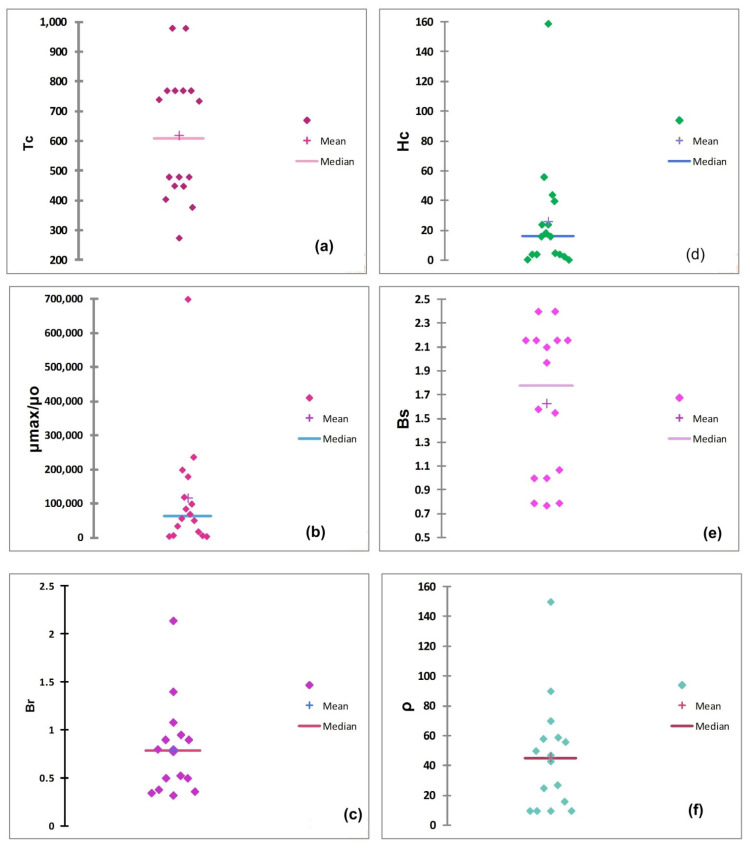
Descriptive data of (**a**) Tc, (**b**) μ_max_/μ_o_, (**c**) Br, (**d**) Hc, (**e**) Bs, (**f**) ρ, for enlisted soft magnetic materials.

To analyze the qualitative data of soft magnetic materials (given in Table 1), XLSTAT and Origin software were employed after the process of data as reported in the literature [21]. The descriptive picture of important physical characteristics of soft magnetic materials, i.e., Tc, μ_max_/μ_o_, Br, Hc, Bs, ρ, is given in Figure 1. It can be seen from the figure that dispersion of data for each characteristic is high, and it is very hard to select any material for a specific application while relying either on mean value or total variance. Coefficients of variance (CV), which are equal to the ratio of standard deviation to mean, is calculated and shown in Table 2. The mean and median values of each physical characteristic are also presented for comparison purpose. The overlapped mean and median values and smaller values of CV of some magnetic properties, i.e., Tc, Br and Bs for enlisted materials (see Table 1 and Table 2 and Figure 1) reveal that these parameters are less important when application relevancy of soft magnetic materials is considered.

The smaller value of CV suggests that Tc is the least important parameter to be considered for selecting the suitable material. The coercive magnetic field ‘Hc’ and magnetic permeability ‘μ_max_/μ_o_’ have higher dispersion of data for the enlisted sixteen magnetic materials (Figure 1b,d), and results in higher CV values, i.e., 1.46 and 1.41, respectively. The coercive magnetic field ‘Hc’ is the ability of a soft magnet to stand against an external magnetic field. As far as applications of soft magnetic materials are concerned, higher values of Hc or values close to transition (of material) from soft to hard magnetic permeability are required. Thus, on a relative scale, the ranking soft magnetic materials can be done while keeping in view the descriptive picture of physical characteristics with the prioritization order given in Table 3.

From Table 3, it is evident the coercive magnetic field (Hc) is the most important and Curie temperature (Tc) is the least important characteristics for selecting any soft magnetic material for a particular application. Our results are in good agreement with the finding reported in the literature [7].

#### 4.1.2. Hierarchy Cluster Analysis

**Figure 2:**
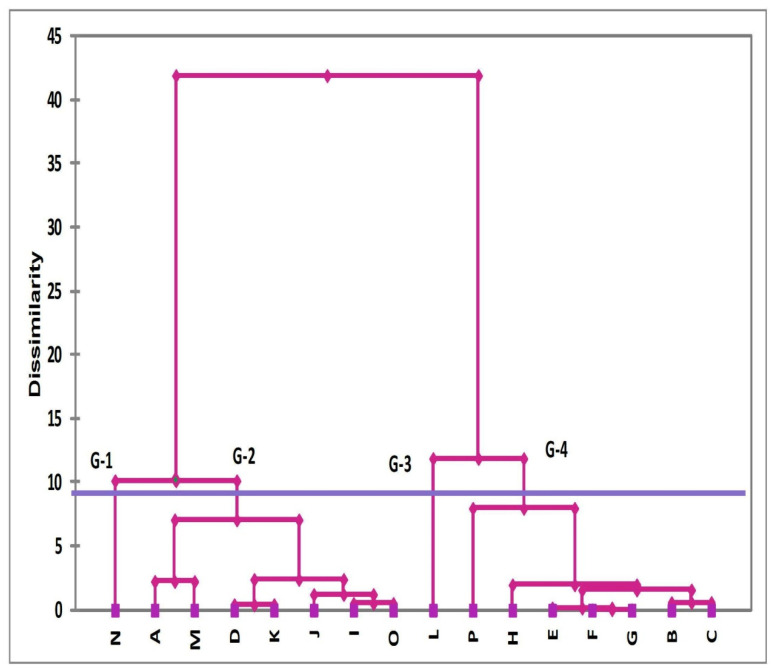
Dendrogram representing the clusters of soft magnetic materials based on the dissimilarities in their physical characteristics.

For further visualization, Cluster analysis (CA) is performed, and results are presented as a dendrogram based on the dissimilarities in properties of enlisted soft magnetic materials. Based on dissimilarities, soft magnetic materials are grouped into four clusters as shown in Figure 2. CA grouped the materials into four clusters, which are labeled as Group 1, 2, 3 and 4. These groups correspond to relatively excellent, good, fair, and bad attributes for soft magnetic materials, respectively. It is evident from the dendrogram shown in Figure 2, that the soft magnetic material Supermalloy, Magnifer 7904 labeled as “N” form G-1 is the best group on the relative scale. The second group consists of seven materials that form the cluster of good magnetic materials and labeled as G-2. The order hierarchy of seven members of this group on relative scale is A→M→D→K→J→I→O. Group 3 of fair materials G-3 has only one material, Permendur 2V labeled as “L”. The poor group is G-4, and it contains seven materials with following hierarchy order P→H→E→F→G→B→C on the relative scale. The priority wise ranking of physical characteristics that is obtained from the descriptive analysis (see Table 3) is used to explain the arrangement of the materials in each cluster, as well as arrangement of the materials within the groups on relative scales. The best material label “N” has a lower value of coercive magnetic field (Hc), and a higher value of relative permeability (μ_max_/μ_o_), thus making G-1 the best. The average values of physical characteristics of all materials in G-2 (except relative permeability) are significantly lower when compared to members present in G-1. However, the values are higher than the members of G-3 and G-4, thus making G-2 good. The materials in G-3 and G-4 are marked as fair and poor on the basis of electrical resistivity (ρ). The value of electrical resistivity ρ for the standalone material in G-3 is lower when compared to the average value of ρ of the G-4 cluster. The electrical resistivity is intrinsic in nature and has no significant correlation with other magnetic properties (as explained in next section) thus playing a significant role in classifications.

#### 4.1.3. Correlation Matrix

For correlation among the magnetic properties, the correlation matrix is calculated, and values are tabulated in Table 4. The correlation matrix, which is calculated from the mean values of magnetic properties of different soft magnetic materials, reflects the dependence of one property to other. Positive correlation implies both a rise and decrease in variable values at the same time. Negative correlation does however suggest the opposite behavior among variables. From Table 4, a strong positive correlation of remanence magnetic induction ‘Br’ and saturation magnetic induction ‘Bs’ with the critical temperature ‘Tc’ is evident. The analysis also reveals the moderate positive correlation between ‘Tc’ and ‘Hc’. In addition to this, a negative correlation between ‘Tc’ with electrical resistivity is also evident. The electrical resistivity has a negative correlation with all physical characteristics except magnetic permeability. All above correlations are true in a physical sense because the hysteresis loss for soft magnetic materials is low. Furthermore, weakness to approximately moderate correlations of three magnetic properties, i.e., corrosive magnetic field ‘Hc’, magnetic permeability μ_max_/μ_o_, and resistivity ρ with other properties is the evidence of the intrinsic nature of these physical characteristics.

### 4.2. Box Plots

**Figure 3:**
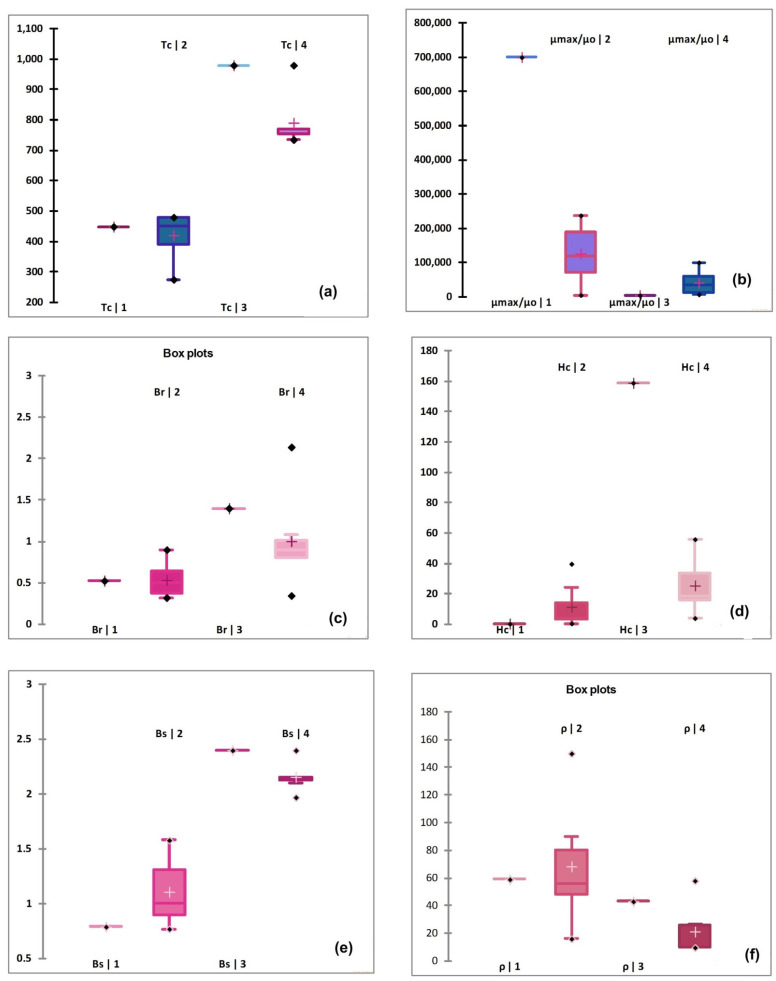
Box plots of (**a**) Tc; (**b**) μ_max_/μ_o_; (**c**) Hc; (**d**) Br; (**e**) Bs; (**f**) ρ, for four clusters based on dendrogram.

For in depth investigation and assessing the accuracy of four clusters, i.e., excellent, good, fair, and poor, the magnetic properties (given in Table 1) are represented as box plots in Figure 3. The member of G-1 has the smallest value of coercive magnetic field Hc and higher value of magnetic permeability. It is therefore the group labeled as excellent based on priority order of physical characteristics as revealed by CV (see Table 3). In addition, the values of Br and Bs for this group are also lower when compared to other clusters, thus complementing the label because of six important physical characteristics of soft magnetic materials [7,22]. Group 2, which is the defined as “good” on the relative scale by cluster analysis, has box plots with a larger spread (Figure 3a–f) for all important magnetic properties. The mean value of Hc, which is close to the desirable value, has a smaller spread of boxes and larger bottom whiskers for ρ and Tc which are the factors responsible for placing the group second on the list. The most and least important priority of Hc and Tc as suggested by descriptive analysis (shown in Table 3) are also in favor of our argument here. The spreads for, μ_max_/μ_o_, Bs and Br boxes for this group are also higher.

Group 3 has only one member labeled as “L” while group 4 consists of seven members. It can be seen that most of the magnetic properties are almost same for both groups, i.e., dissimilarities among characteristics are negligible but the dendrogram classified group 3 as a fair material group, labeling the remaining group as poor, i.e., group 4. The higher values of Hc and the lowest values of μ_max_/μ_o_ are the reason for classifying groups as excellent to poor on the relative scale as evident from the box plots (see Figure 3). The electrical resistivity ρ is the physical characteristic in addition to Hc and μ_max_/μ_o_ for further classification of materials as fair and poor. The lowest mean values and the larger spread of data for resistivity ρ that is evident from box plots (see Figure 3f) are the reasons for making the distinction among fair and poor magnetic materials.

### 4.3. Principal Component Analysis (PCA)

The Eigen values (obtain after performing PCA) corresponding to the six physical characteristics of soft magnetic materials are listed in the Table 5. Only the first three sets are sufficient to explain the information contained in the original data. Moreover, the Eigen values are > 0.5, and the percentage variance of first three Eigen values is 86%, which is sufficient for using the data sets for classification purposes [20]. The percentages of variances confirm that one can apply the PCA with confidence for analysis of data. The Eigen values correspond to six physical characteristics, i.e., for Tc, μ_max_/μ_o_, Br, Hc, Bs and ρ are 3.488, 1.043, 0.684, 0.584, 0.169, and 0.032 respectively. The larger Eigen value for Tc suggests that the dispersion in Tc values is higher for enlisted magnetic materials (see Table 1 for details). The main reasons for higher Eigen value for Tc values are the mean, median and standard deviation in the data for enlisted materials. These values of Tc vary from 450 °C to 980 °C with mean and median values equal to 619.5 °C and 607.5 °C, respectively. The standard deviation in the data is ~± 211 °C (as evident from descriptive analysis and box plots of four groups). In addition to this, large dispersion in Tc values is also the reason for making this magnetic property the least important in the list. For μ_max_/μ_o_, the significant higher value of magnetic permeability for Supermalloy, Magnifer 7904, i.e., 700,000 is responsible for obtaining the Eigen value 1.043. The equal distribution of electrical resistivity ρ values from average values and having almost the same mean and median values is the reason for obtaining the lowest Eigen value, i.e., 0.032 for this characteristic. The box plot for electrical resistivity ρ (shown in Figure 3f) also complements the PCA analysis and favors the lowest Eigen value of resistivity (ρ).

According to well documented PCA theory [21], the characteristics loadings are the representation of correlation among active variables and coefficients. These loadings are the projections of magnetic characteristics on the principal component’s (PCs) axis. The characteristic loading among the variables/properties for soft magnetic materials are tabulated in Table 5 and classified as strong, moderate and weak. If a characteristic loading > 0.75, then it is considered as “strong”, characteristic loading between 0.75–0.50 corresponds to “moderate” and values between 0.50–0.30 correspond to “weak” characteristics loading. Furthermore, positive and negative loadings are the representation of direct proportions and indirect proportions, respectively [23].

The PC-1 that defines 58.13% of total variance has strong positive characteristic Loadings, i.e., >0.75 for Tc & Bs, moderate (positive) loadings for Br & Hc and moderate (negative) loadings for μ_max_/μ_o_ and ρ. The characteristics which have larger dispersion of data, higher values of standard deviations and least importance (in nature), i.e., Tc and Bs (see Table 2), are responsible for defining this PC. The almost same loading values of Hc and μ_max_/μ_o_ with a negative correlation among each other endorse the precision of this PC. In addition, the characteristic loadings given in Table 5 also reveal the fact that this PC is strongly affected by the dependent magnetic properties. The major contributing characteristics for PC-2 are Hc, ρ, and μ_max_/μ_o_, respectively. All three characteristics have moderate loadings with the only difference being that μ_max_/μ_o_ has negative loadings [24]. The weak loadings of other three characteristics, i.e., Tc, Br and Bs suggest that PC-2 (which defines 17% of total variance) is dominated by those magnetic characteristics that are intrinsic in nature.

**Figure 4:**
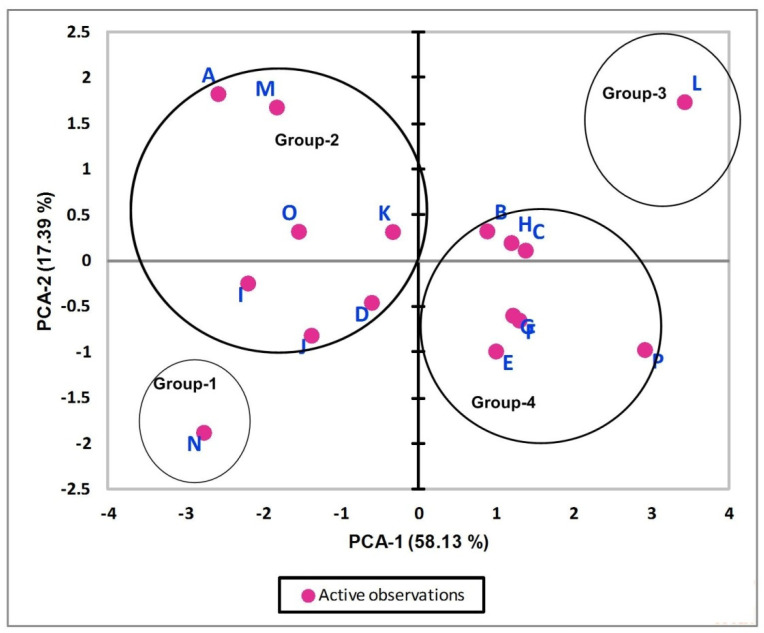
Loadings of magnetic characteristics of soft magnetic materials on PCA-1 and PCA-2.

**Figure 5:**
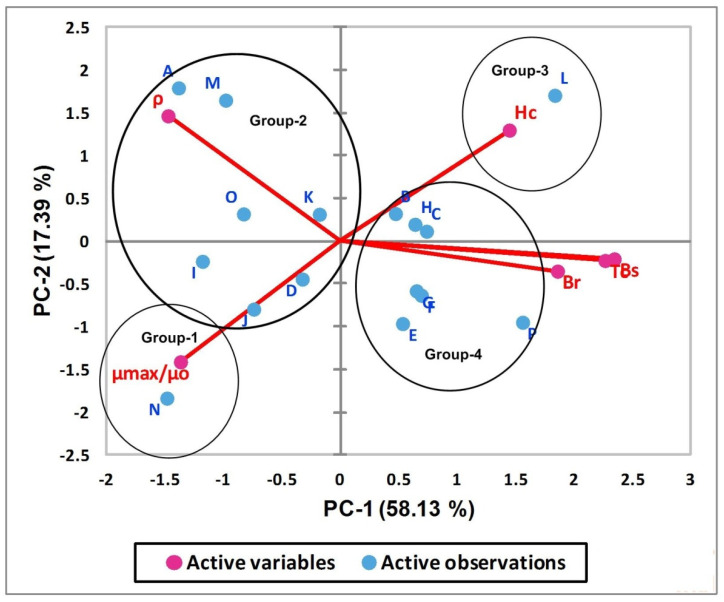
Bipolar PCA plot representing active variables and observations on the first two PCs space.

The characteristic loadings for enlisted soft magnetic materials on the first two principal axis are represented in Figure 4. Groups with standalone magnetic materials, i.e., G-1 and G-3, have strong negative loadings and strong positive loadings on PC-1 and PC-2, respectively. This is true as far as physics of magnetic characteristic is concerned because the lowest value of Hc and the highest values of μ_max_/μ_o_ are required for the best alternative. The higher value of μ_max_/μ_o_, lower value of Hc and the moderate positive loading of resistivity ρ (as evident from Table 5) are the major reasons for placing the material with label N in third quadrant (see Figure 4). The materials that form group 2 in the cluster analysis show weak to moderate negative loadings on PC-1, and this is true for all materials belonging to this group as far as loadings on PC-1 are concerned (see Figure 4 for detail). In addition to this, the materials which have strong negative loadings on PC-1 also have strong positive loadings on PC-2 which is confirmation of the agreement of PCA results with cluster analysis outcomes. The classification of enlisted materials into excellent, good, fair, poor groups and prioritization ordering of materials on a relative scale, i.e., A→M→D→K→J→I→O, is also confirmed by PCA. The hieratic order of A, M, I, O is clear as A and M have strong negative and positive loadings on PC-1 and PC-2, respectively. The materials labelled as I and O have moderate negative loadings on PC-1 and weak loadings on PC-2. The complete picture of prioritization order is clearer while looking at the bipolar chart given in Figure 5. Group 3 consists of only one material labeled as L and has strong positive loadings on PC-1 and PC-2, respectively. The strong positive loadings on PC-1 means this group has the lowest Hc which is the most important characteristic in the list, thus labelling this group as fair. Group 4 (except material labeled as P) has weak to moderate positive loading on PC-1. In addition to this, it has weak loadings (positive and/or negative) on PC-2, thus labelling it as poor. The prioritization order on the relative scale is also evident from Figure 4 and in agreement with the findings of the cluster analysis, i.e., P→H→E→F→G→B→C.

The bipolar plot of the magnetic characteristics for enlisted materials in the first two PCs space is shown in Figure 5. It clearly depicts the influence of six physical characteristics to organize the eighteen materials in four groups as excellent, good, fair and poor. Group 1 (which has strong negative loadings on PC-1 and PC-2) is strongly influenced by the magnetic permeability, i.e., μ_max_/μ_o_. The resistivity ρ is the major responsible factor for labelling group 2 as good, while the combine influence of ρ, μ_max_/μ_o_, and Hc is responsible for defining the prioritization order of the materials within the group. The relative overture of hierarchy order within the groups (obtained from cluster analysis) seems logical while considering the results of bipolar chart and precedence of physical characteristics given in Table 3. The strong positive loadings on PC-1 and PC-2 along with dominant influence of coercive magnetic field Hc (see Figure 5) is responsible for group 3. Furthermore, Bs, Br, and Tc are the major responsible factors for arranging the remaining seven materials in group 4. The preference listing of magnetic characteristics, i.e., Hc > μ_max_/μ _o_> ρ > Br > Bs > Tc, is the reason for the members of group 4 to be considered as poor.

**Figure 6:**
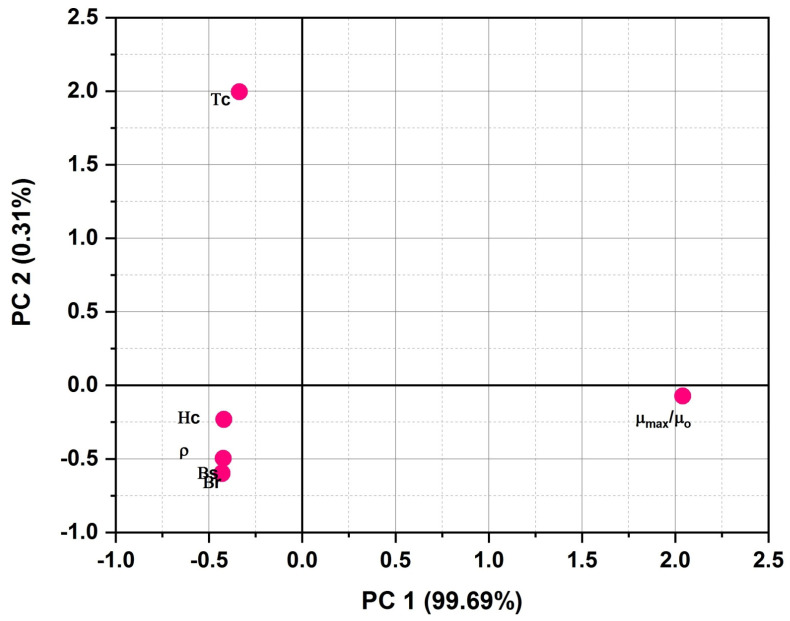
PCA plot of magnetic characteristics on the first two PCs space.

Finally, Figure 6 which represents the loadings of magnetic characteristics (considered for classification) on the first two PCs axis reveals the following significant trends:

- PC-1 defines the 99.69% while PC-2 defines only 0.31% of total variance, which means that loadings on PC-1 clearly define the importance of active variables;
- μ_max_/μ_o_ has strong positive loadings on PC-1 and weak negative (among all) loading on PC-2;
- All other characteristics are inversely related with the μ_max_/μ_o_ with only a difference in loading values on PC-2;
- As far as other physical characteristics are concerned, all characteristics (except μ_max_/μ_o_) have strong negative loadings on PC-1;
- Hc, ρ, Br, Bs have weak negative loadings, and Tc has moderate positive loading on PC-2.
- On the basis of the above trends, it can be said conclusively that the most important magnetic properties in case of soft magnetic material are the magnetic permeability μ_max_/μ_o_ and coercive magnetic field Hc, respectively.

### 4.4. Application of Graph Theory and Matrix (GTMA) Approach for Magnetic Material Selection

For the selection of optimum soft magnetic material, the choice and the ranking of material selection criteria plays an important role. Attributes for the selection process are already ranked by using multivariate analysis as explained in Section 3.1. With the help of PCA soft magnetic materials are classified into four groups termed as excellent, good, fair and poor on the basis of selected beneficial and non-beneficial factors. However, the classification of materials into four groups is not sufficient. Therefore, graph theory is applied to rank the materials based on selected criteria and their relative importance. Briefly, graph theory is the study of graphs, which are mathematical models used to construct pair wise relationships between objects. For material selections this graph constructs the interrelation between different material selection attributes. In this framework, a graph is made up of vertices also defined as nodes that represent the material selection factors, and these nodes are connected by edges also called links or lines that show the relative importance’s among the factors.

#### 4.4.1. Schematic Representation of Attributes

**Figure 7:**
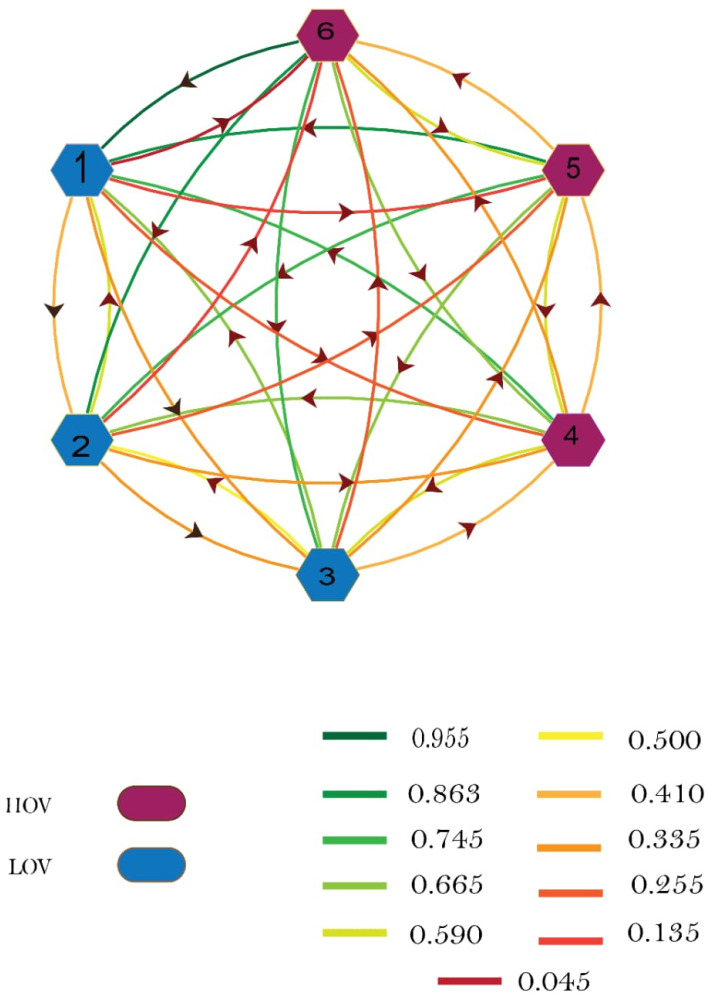
Graphical representation of relative importance between attributes.

The material assessment graph as shown in Figure 7 is plotted using Mathematical software by considering six different material selection factors. As there are six material selection factors, there are six corresponding nodes labeled as 1, 2, 3, 4, 5, and 6, respectively. The graph displays the arrow from node 1 to 2 and likewise from 2 to 1, the color of each arrow represents the relative importance of one parameter to another. Furthermore, higher obligatory value (HOV) and lower values obligatory value (LOV) attributes are also represented with colors (see Figure 7). Maximum relative permeability is more important than Curie temperature in the process of material selection, but Curie temperature is also important, though less important than maximum relative magnetic permeability, so in both cases, relative importance is present between the two attributes. Similarly, interrelation is present between other factors. However, this graph is only a visual representation of material selection factors and their relative importance. For analysis, an adjacency matrix of the graph is written, which is then used for figuring out the material suitability index for each enlisted material.

#### 4.4.2. Material Suitability Index

The material suitability index is the numerical value of the material selection factors function. There are six magnetic material selection attributes so there will be a 6 × 6 matrix with diagonal elements R_i_ and off diagonal r_ij_. For each material, all factors (i.e., Ri) and their relative importance (i.e., rij) are considered for establishing the decision matrix for each material. This decision matrix is then used for calculating the suitability index.

$$A\;=\;[\begin{array}{ccccccccc} Parameter & F_{1} & F_{2} & F_{3} & F_{4} & F_{5} & F_{6} & F_{7} & F_{8}\\ F_{1} & R_{1} & r_{12} & r_{13} & r_{14} & r_{15} & r_{16} & r_{17} & r_{18}\\ F_{2} & r_{21} & R_{2} & r_{23} & r_{24} & r_{25} & r_{26} & r_{27} & r_{28}\\ F_{3} & r_{31} & r_{32} & R_{3} & r_{34} & r_{35} & r_{36} & r_{37} & r_{38}\\ F_{4} & r_{41} & r_{42} & r_{43} & R_{4} & r_{45} & r_{46} & r_{47} & r_{48}\\ F_{5} & r_{51} & r_{52} & r_{53} & r_{54} & R_{5} & r_{56} & r_{57} & r_{58}\\ F_{6} & r_{61} & r_{62} & r_{63} & r_{64} & r_{65} & R_{6} & r_{67} & r_{68}\\ F_{7} & r_{71} & r_{72} & r_{73} & r_{74} & r_{75} & r_{76} & R_{7} & r_{78}\\ F_{8} & r_{81} & r_{82} & r_{83} & r_{84} & r_{85} & r_{86} & r_{87} & R_{8} \end{array}]$$

#### 4.4.3. Representation of Matrix

To calculate the diagonal elements R_i,_ the normalized values of quantitative data is considered. To figure out the normalized values R_i,_ figuring out the beneficial and non-beneficial factors must be the first step. There are six attributes in the present case, and all are already ranked according to their importance in Table 3. The first three attributes (Hc, μ_max_/μ_o_, ρ) are considered as beneficial and the last three as non-beneficial (Br, Bs, Tc). For beneficial factors, the higher values are preferable, and for non-beneficial factors, lower values are required. First consider beneficial factors and then normalize the data by using the relation ***v_i_/v_j_*,** where *v_i_* is the factor value for the i^th^ alternative, and v_j_ is the factor value for the j^th^ alternative, it also has the highest factor value among the alternatives studied. For non-beneficial factors, **v_j/_v_i_** is used for calculating the normalized values assigned to the alternatives. In this respect, v_j_ is the factor value for the j^th^ alternative, which has the lowest factor measure among the alternatives investigated. The normalized values are represented in Table 6.

The next step is to find the relative importance, i.e., r_ij_, which are off diagonal elements of the decision matrix. For this, an 11-point fuzzy scale along with rankings of attributes that are already performed by statistical analysis (see Table 3) is used to assign verbal relative importance to corresponding attribute. The 11-point fuzzy logic scale for converting the verbal scale into number is tabulated in Table 7.

The decision matrix in Table 8 shows that critical temperature T_C_ is slightly less important than B_S_. Similarly, the relative importance between Tc and Br is 0.335, which indicates that Tc is less important than Br. According to the ranking of attributes with respect to their relative importance, coercive magnetic field Hc is the most important attribute so the relative importance between T_C_ and Hc has a numeric value of 0.135 that shows that critical temperature Tc is extremely less important than coercive magnetic field Hc. Likewise, Hc is extremely more important than Tc, so the relative importance between them has numerical value of 0.865. The values on the diagonal indicate the relation between the same attributes, and off-diagonal values show the relative importance between different attributes. Each material’s ranking index is computed while using the following equation:

Ranking Index = Per (A)

where the Per (A) is the determinant of a matrix (a) with all positive entries in the calculation.

The above expression is the full representation for the material selection problem under consideration, as it considers the presence of all elements and all conceivable relative importance relationships between them. By using the adjacency matrix and the normalized data of each material, the permanent function for each material is calculated and the suitability index is given. The material with the highest value of suitability index is considered the best material among all alternatives.

$$\begin{array}{cc} \mathrm{Per}\;\left(A\right)=\;\overset{N}{\underset{i=1}{\prod }}R_{i} & \;+\overset{N-1}{\underset{i=1}{\sum }}\overset{N}{\underset{j=i+1}{\sum }}\ldots \times \overset{N}{\underset{N=t+1}{\sum }}(r_{ij}r_{ji})\times R_{k}R_{l}R_{m}R_{n}R_{o}\ldots R_{t}R_{N\;}\\ & +\overset{N-2}{\underset{i=1}{\sum }}\overset{N-1}{\underset{j=i+1}{\sum }}\overset{N}{\underset{k=j+1}{\sum }}\ldots \times \overset{N}{\underset{N=t+1}{\sum }}(r_{ij}r_{jk}r_{ki}+r_{ik}r_{kj}r_{ji})\\ & \times R_{k}R_{l}R_{m}R_{n}R_{o}\ldots R_{t}R_{N}\\ & +(\overset{N-3}{\underset{i=1}{\sum }}\overset{N}{\underset{j=i+1}{\sum }}\overset{N-1}{\underset{k=j+1}{\sum }}\overset{N}{\underset{l=j+2}{\sum }}\ldots \times \overset{N}{\underset{N=t+1}{\sum }}(r_{ij}r_{jk}r_{kl}r_{li}+r_{il}r_{lk}r_{kj}r_{ji})\\ & \times R_{k}R_{l}R_{m}R_{n}R_{o}\ldots R_{t}R_{N})\\ & +(\overset{N-2}{\underset{i=1}{\sum }}\overset{N-1}{\underset{j=i+1}{\sum }}\overset{N}{\underset{k=j+1}{\sum }}\overset{N-1}{\underset{l=1}{\sum }}\overset{N}{\underset{m=l+1}{\sum }}\ldots \times \overset{N}{\underset{N=t+1}{\sum }}(r_{ij}r_{jk}r_{ki}\\ & +r_{ik}r_{kj}r_{ji})\left(r_{lm}r_{ml}\right)\times R_{k}R_{l}R_{m}R_{n}R_{o}\ldots R_{t}R_{N}\\ & +\;\overset{N-4}{\underset{i=1}{\sum }}\overset{N-1}{\underset{j=i+1}{\sum }}\overset{N}{\underset{k=i+1}{\sum }}\overset{N}{\underset{l=i+1}{\sum }}\overset{N}{\underset{m=j+1}{\sum }}\ldots \times \overset{N}{\underset{N=t+1}{\sum }}(r_{ij}r_{jk}r_{kl}r_{lm}r_{li}\\ & +r_{im}r_{ml}r_{ik}r_{kj}r_{jii})\times R_{k}R_{l}R_{m}R_{n}R_{o}\ldots R_{t}R_{N})\\ & +(\overset{N-3}{\underset{i=1}{\sum }}\overset{N-1}{\underset{j=i+1}{\sum }}\overset{N}{\underset{k=j+1}{\sum }}\overset{N}{\underset{l=j+1}{\sum }}\overset{N-1}{\underset{m=l}{\sum }}\overset{N}{\underset{n=m+1}{\sum }}\ldots \times \overset{N}{\underset{N=t+1}{\sum }}(r_{ij}r_{jk}r_{kl\;}r_{li}\\ & +r_{il}r_{ik}r_{kj}r_{ji})\left(r_{mn}r_{nm}\right)\times R_{k}R_{l}R_{m}R_{n}R_{o}\ldots R_{t}R_{N}\\ & +\;\overset{N-5}{\underset{i=l}{\sum }}\overset{N-1}{\underset{j=i+1}{\sum }}\overset{N}{\underset{k=j+1}{\sum }}\overset{N-2}{\underset{l=1}{\sum }}\overset{N-1}{\underset{m=l+1}{\sum }}\overset{N}{\underset{n=m+1}{\sum }}\ldots \times \overset{N}{\underset{N=t+1}{\sum }}(r_{ij}r_{jk}r_{kl}\\ & +r_{ik}r_{kj}r_{jii})(r_{lm}r_{mn}r_{nl}+r_{ln}r_{nm}r_{ml})\times R_{k}R_{l}R_{m}R_{n}R_{o}\ldots R_{t}R_{N})\\ & +(\overset{N-5}{\underset{i=1}{\sum }}\overset{N}{\underset{j=i+1}{\sum }}\overset{N-3}{\underset{k=i+1}{\sum }}\overset{N}{\underset{l=i+1}{\sum }}\overset{N-1}{\underset{m=k+1}{\sum }}\overset{N}{\underset{n=k+2}{\sum }}\ldots \\ & \times \overset{N}{\underset{N=t+1}{\sum }}(r_{ij}r_{ji})(r_{kl\;}r_{lk})\left(r_{mn}r_{nm}\right)\times R_{k}R_{l}R_{m}R_{n}R_{o}\ldots R_{t}R_{N}\\ & +\;\overset{N-5}{\underset{i=l}{\sum }}\overset{N-1}{\underset{j=i+1}{\sum }}\overset{N}{\underset{k=i+1}{\sum }}\overset{N}{\underset{l=i+1}{\sum }}\overset{N}{\underset{m=i+1}{\sum }}\overset{N}{\underset{n=j+1}{\sum }}\ldots \times \overset{N}{\underset{N=t+1}{\sum }}(r_{ij}r_{jk}r_{kl}\\ & r_{ik}r_{kj}r_{jii})(r_{lm}r_{mn}r_{nl}+r_{ln}r_{nm}r_{ml})\times R_{k}R_{l}R_{m}R_{n}R_{o}\ldots R_{t}R_{N}) \end{array}$$

Table 9 indicates that Supermalloy, Magnifer 7904 is the optimal choice among the enlisted alternatives. The selection is made by considering the application specific magnetic properties of magnetic material. The results are also in accordance with the multivariate statistical analysis. According to multivariate analysis, Group 1 containing Supermalloy, Magnifer 7904 labelled as N (which has strong negative loadings on PC-1 and PC-2) is strongly influenced by the magnetic permeability (i.e., μ_max_/μ_o)_ and is termed as excellent. This material is also ranked as number 1 according to the suitability index value calculated while using the concepts of graph theory. According to PCA, group 2 consists of materials labeled as M, A, I, O, J, D, K and are termed as good due to strong influence of resistivity ρ, while the combined influence of ρ, μ_max_/μ_o_ and Hc is responsible for the prioritization ordering of the materials within the group. The parametric graph theory and matrix approach (GTMA) also ranked the members of this group from 2nd–6th to choose the next best options after Supermalloy, Magnifer 7904. Group 3 contains only one material labeled as L, termed as fair and lies at 7th rank according to GTMA. All the remaining materials lie in group 4 and are termed as poor according to PCA which is further confirmed by GTMA.

In summary, the results reported in this study reveal that multivariate statistics provides a simple and robust alternative for selecting the suitable magnetic material. It provides the user flexibility to avoid complex mathematical formulations used in other MCDM. In addition to this, the systematic nature of the method and authenticity (as evident from literature [10,11] and confirmed with GTMA model) makes it more appealing for other multi-attribute material selection applications.

## 5. Conclusions

Cluster analysis revealed that soft magnetic materials can be classified in excellent, good, fair and poor materials on the basis of physical characteristics. Descriptive statistics and CV defined the prioritization order of magnetic characteristics as Hc > μ_max_/μ_o_ > ρ > Br > Bs > Tc, and this precedence of magnetic properties was responsible to classify the materials into four clusters. The relative permeability μ_max_/μ_o_, coercive magnetic field Hc, and electrical resistivity were responsible for explaining ~99.90% of total variance as confirmed from principal component analysis. All groups were significantly influenced by μ_max_/μ_o_ and Hc. In addition to this, combined influence of electrical resistivity ρ with μ_max_/μ_o_ and Hc was responsible for further organizing the materials in group 2. Similarly, Br, Bs, and Tc played their roles for organizing the materials within group 4. Furthermore, principal component analysis confirmed the fact that the highest value of relative permeability and lowest value of Hc was required for them most suitable soft magnetic material.

After prioritizing the attributes, GTMA is applied to identify the best material among enlisted alternatives. For this purpose, the suitability index helps in ranking the soft magnetic materials according to their importance based on selected attributes/magnetic properties. The results revealed that they are in consonance with the principal component analysis. According to these results, the soft magnetic material named as Supermalloy, Magnifer 7904 labelled as L, identified as excellent and ranked first place by GTMA thus complementing the classification of multivariate statistical analysis. Although, the proposed simple and robust technique for material selection relies on the application’s specific physical characteristics, it can be used for other materials used for industrial application.

## Figures and Tables

**Table 1:** List of soft magnetic materials with their physical properties [15,16].

Materials	Labels	Composition (w/wt.%)	Tc (°C)	μ_max_/μ_o_	Br (T)	Hc (A.m^−1^)	Bs (T)	ρ (μΩcm)
Alfenol 16	A	84Fe–16Al	450	85,500	0.38	2.59	0.79	150
Ferrosilicon	B	96Fe–4Si	735	18,500	1.08	24	1.97	58
Ferrosilicon	C	99Fe–1Si	740	7700	0.95	44	2.1	25
Hypernik V	D	51Fe–49Ni	480	180,000	0.9	4.8	1.55	47
Iron (H_2_ reduced)	E	99.9Fe	770	100,000	0.8	4	2.158	9.71
Iron (electrolytic)	F	99.9Fe	770	51,250	0.9	18.4	2.158	9.71
Iron (carbonyl)	G	99.9Fe	770	35,000	0.8	16	2.158	9.71
Iron (Armco)	H	99.99Fe	770	7000	0.345	56	2.158	9.71
MuMetal	I	77Ni–16Fe–5Cu–2Cr	405	237,500	0.32	0.5	0.77	56
Permalloy 78	J	78.5Ni–21.5Fe	378	200,000	0.5	4	1.07	16
Permalloy 45	K	55Fe–45Ni	480	57,500	0.775	24	1.58	50
Permendur 2V	L	49Fe–49Co–2V	980	4500	1.4	159	2.4	43
Rhometal	M	64Fe–36Ni	275	5000	0.36	39.79	1	90
Supermalloy,	N	79Ni–15Fe–5Mo–	449	700,000	0.525	0.35	0.79	59
Magnifer 7904		0.5Mn						
Sendust	O	85Fe–10Si–5Al	480	120,000	0.5	3.98	1	70
Supermendur	P	49Fe–49Co–2V	980	70,000	2.14	16	2.4	27

**Table 2:** Mean, Median and Coefficient of variance of physical characteristics of soft magnetic materials.

Properties	Mean	Median	CV
Tc	619.5	607.500	0.34
μ_max/_μ_o_	117,465.6	63,750.000	1.41
Br	0.792	0.788	0.58
Hc	26.088	16.000	1.46
Bs	1.628	1.775	0.37
ρ	45.615	45.000	0.79

**Table 3:** Priority of physical characteristics for ranking the soft magnetic materials.

Order	1	2	3	4	5	6
CV	1.46	1.41	0.79	0.58	0.37	0.34
Property	Hc	μ_max_/μ_o_	Ρ	Br	Bs	Tc

**Table 4:** Pearson correlation matrix of physical characteristics.

Physical Characteristics	Br	Tc	Bs	ρ	Hc	μ_max_/μ_o_
Br	1					
Tc	0.758	1				
Bs	0.697	0.922	1			
ρ	−0.319	−0.532	−0.665	1		
Hc	0.318	0.509	0.487	−0.118	1	
μ_max_/μ_o_	−0.251	−0.401	−0.557	0.108	−0.394	1

**Table 5:** Characteristic loading and Eigen values of magnetic properties.

	PCA-1	PCA-2	PCA-3
Tc	0.941	−0.093	0.149
μ_max_/μ_o_	−0.569	−0.586	0.474
Br	0.772	−0.145	0.475
Hc	0.600	0.540	0.199
Bs	0.973	−0.086	−0.115
Ρ	−0.613	0.610	0.398
Eigenvalue	3.488	1.043	0.684
Variability (%)	58.132	17.389	11.395
Cumulative %	58.132	75.521	86.916

**Table 6:** Normalized data of soft magnetic material selection attributes.

Materials	Labels	Composition (w/wt.%)	Tc (˚C)	mmax/mo	Br (T)	Hc (A.m^−1^)	Bs (T)	r (mWcm)
Alfenol 16	A	84Fe–16Al	0.611	0.122143	0.842105	0.016289	0.974684	1
Ferrosilicon	B	96Fe–4Si	0.3474	0.3474	0.296296	0.150943	0.390863	0.38667
Ferrosilicon	C	99Fe–1Si	0.37162	0.011	0.33684	0.27673	0.36667	0.166667
Hypernik V	D	51Fe–49Ni	0.5729	0.257142	0.35556	0.030189	0.496774	0.31333
Iron (H_2_ reduced)	E	99.9Fe	0.357	0.142857	0.4	0.025157	0.356812	0.064733
Iron (electrolytic)	F	99.9Fe	0.357	0.07321	0.355556	0.115723	0.356812	0.064733
Iron (carbonyl)	G	99.9Fe	0.357	0.05	0.4	0.100629	0.356812	0.064733
Iron (Armco)	H	99.99Fe	0.357	0.01	0.92754	0.3522	0.356812	0.064733
MuMetal	I	77Ni–16Fe–5Cu–2Cr	0.679	0.3393	1	0.003145	1	0.373333
Permalloy 78	J	78.5Ni–21.5Fe	0.7275	0.2857	0.64	0.002516	0.719626	0.106667
Permalloy 45	K	55Fe–45Ni	0.5729	0.0821	0.4129	0.150943	0.320833	0.333333
Permendur 2V	L	49Fe–49Co–2V	0.2806	0.00643	0.22857	1	0.320833	0.286667
Rhometal	M	64Fe–36Ni	1	0.007143	0.88889	0.250252	0.77	0.6
Supermalloy,	N	79Ni–15Fe–5Mo–	0.6125	1	0.609524	0.002201	0.974684	0.393333
Magnifer 7904		0.5Mn						
Sendust	O	85Fe–10Si–5Al	0.5729	0.17143	0.64	0.025031	0.77	0.466667
Supermendur	P	49Fe–49Co–2V	0.2806	0.1	0.149533	0.100629	0.3208	0.18

**Table 7:** 11-point fuzzy logic scale.

Descriptive Statement	Quantitative Weight
One parameter has exceptionally low importance when compared to others.	0.045
One parameter has extremely low importance when compared to others.	0.135
One parameter has very low importance when compared to others.	0.255
One parameter has low importance when compared to others.	0.335
One parameter has slightly below equal importance when compared to others.	0.41
One parameter has equal importance when compared to others.	0.5
One parameter has slightly above equal importance when compared to others.	0.59
One parameter has high importance when compared to others.	0.665
One parameter has very high importance when compared to others.	0.745
One parameter has extremely high importance when compared to others.	0.865
One parameter has exceptionally high importance when compared to others.	0.955

**Table 8:** Decision matrix from relative importance of material selection factor (11-point scale).

Property	Tc	Bs	Br	ρ	μ_max_/μ_o_	Hc
Tc	R_1_	0.41	0.335	0.335	0.255	0.135
Bs	0.59	R_2_	0.41	0.255	0.135	0.045
Br	0.665	0.59	R_3_	0.41	0.335	0.255
ρ	0.665	0.745	0.59	R_4_	0.41	0.335
μ_max_/μ_o_	0.745	0.865	0.665	0.59	R_5_	0.41
Hc	0.865	0.955	0.745	0.665	0.59	R_6_

**Table 9:** Suitability Index and Ranking of enlisted Soft magnetic materials.

Materials	Labels	Composition (w/wt.%)	Tc (˚C)	µ_max_/µ_o_	Br (T)	Hc (A.m^−1^)	Bs (T)	r (mWcm)	Permanents of Matrix
Alfenol 16	A	84Fe–16Al	450	85,500	0.38	2.59	0.79	150	6.46209
Ferrosilicon	B	96Fe–4Si	735	18,500	1.08	24	1.97	58	3.84094
Ferrosilicon	C	99Fe–1Si	740	7700	0.95	44	2.1	25	3.2374
Hypernik V	D	51Fe–49Ni	480	180,000	0.9	4.8	1.55	47	3.93485
Iron (H_2_ reduced)	E	99.9Fe	770	100,000	0.8	4	2.158	9.71	3.115
Iron (electrolytic)	F	99.9Fe	770	51,250	0.9	18.4	2.158	9.71	3.05412
Iron (carbonyl)	G	99.9Fe	770	35,000	0.8	16	2.158	9.71	3.06157
Iron (Armco)	H	99.99Fe	770	7000	0.345	56	2.158	9.71	4.04846
MuMetal	I	77Ni–16Fe–5Cu–2Cr	405	237,500	0.32	0.5	0.77	56	6.49842
Permalloy 78	J	78.5Ni–21.5Fe	378	200,000	0.5	4	1.07	16	4.68784
Permalloy 45	K	55Fe–45Ni	480	57,500	0.775	24	1.58	50	3.70229
Permendur 2V	L	49Fe–49Co–2V	980	4500	1.4	159	2.4	43	3.79996
Rhometal	M	64Fe–36Ni	275	5000	0.36	39.79	1	90	6.48505
Supermalloy, Magnifier 7904	N	79Ni–15Fe–5Mo–0.5Mn	449	700,000	0.525	0.35	0.79	59	7.0907
Sendust	O	85Fe–10Si–5Al	480	120,000	0.5	3.98	1	70	4.86904
Supermendur	P	49Fe–49Co–2V	980	70,000	2.14	16	2.4	27	2.79293
